# Is Hunting Still Healthy? Understanding the Interrelationships between Indigenous Participation in Land-Based Practices and Human-Environmental Health

**DOI:** 10.3390/ijerph110605751

**Published:** 2014-05-28

**Authors:** Ursula King, Christopher Furgal

**Affiliations:** 1 National Centre for Epidemiology and Population Health, College of Medicine, Biology and Environment, Australian National University, Canberra, ACT 0200, Australia; 2 Indigenous Environmental Studies Program, Trent University, Peterborough, ON K9J 7B8, Canada; E-Mail: chrisfurgal@trentu.ca

**Keywords:** Indigenous, land-based practices, health, environment, transdisciplinary

## Abstract

Indigenous participation in land-based practices such as hunting, fishing, ceremony, and land care has a long history. In recent years, researchers and policy makers have advocated the benefits of these practices for both Indigenous people and the places they live. However, there have also been documented risks associated with participation in these activities. Environmental change brought about by shifts in land use, climate changes, and the accumulation of contaminants in the food chain sit alongside equally rapid shifts in social, economic and cultural circumstances, preferences and practices. To date, the literature has not offered a wide-ranging review of the available cross-disciplinary or cross-ecozone evidence for these intersecting benefits and risks, for both human and environmental health and wellbeing. By utilising hunting as a case study, this paper seeks to fill part of that gap through a transdisciplinary meta-analysis of the international literature exploring the ways in which Indigenous participation in land-based practices and human-environmental health have been studied, where the current gaps are, and how these findings could be used to inform research and policy. The result is an intriguing summary of disparate research that highlights the patchwork of contradictory understandings, and uneven regional emphasis, that have been documented. A new model was subsequently developed that facilitates a more in-depth consideration of these complex issues within local-global scale considerations. These findings challenge the bounded disciplinary and geographic spaces in which much of this work has occurred to date, and opens a dialogue to consider the importance of approaching these issues holistically.

## 1. Introduction

A feature of the Second International Decade of the World’s Indigenous People (2005 to 2014) has been the promotion by human-rights agencies of Indigenous (“Indigenous” is the term used in this paper to describe Inuit, Aboriginal, First Nation and other First Peoples, except when referring to a specific group, in which case the term preferred by that group is used) peoples’ knowledge’s and participation in land-based practices [1,2]. As part of a wider sustainable development agenda, the key focus has been the contribution Indigenous people make to improvements in ecosystem health through “traditional” land-based (land-based practices encompass both land and sea/waterway/seaice activities engaged in by Indigenous people, and include hunting, fishing, gathering/collecting, natural resource management, plant cultivation, and ceremony) activities. By extension, there has been growing interest in how these activities could be harnessed in human health policy and programs to improve Indigenous health and wellbeing [3,4,5]. Central to the call for promotion of these activities has been the assertion that both Indigenous health, and the health of the places Indigenous people live, could be improved by ongoing and supported Indigenous participation in activities that connect them. It is an appealing argument, from both a human and ecosystem health perspective. 

Considered a staple part of many remote Indigenous people’s lives, the historical image of people living on and from the land through activities such as hunting, fishing and gathering of local resources is still seen today as a culturally important, and fundamentally healthy, way of life. On a background of, often substantial, Indigenous health inequity compared to non-Indigenous members of the same state/country, and increasing concerns about the impacts on Indigenous health of environmental contamination and degradation from competing land uses, the assertion that “hunting is healthy” has gathered momentum. Hunting is used here as an illustrative case because of its predominance in the international literature as a substantive land-based activity practised by numerous Indigenous peoples around the world, and taken to mean hunting, fishing and gathering of local resources. Led by calls from health researchers, and echoing long expressed Indigenous voices, current thinking supports encouragement of Indigenous participation in land-based practices (LBP), such as hunting, as a means of promoting health—of both people and place. To date, no papers appear to have been published that offer an in-depth review of the available international, cross-disciplinary evidence drawn from various ecozones to support this promotion. By drawing on techniques used within transdisciplinary health research, this paper seeks to fill that gap by identifying, gathering and critically analysing the international literature to identify the available evidence, and any omissions that may exist. 

### 1.1. Transdisciplinary Approaches to Human Health

Transdisciplinary approaches to thinking about human health are increasingly being seen as a way of unifying understandings, and developing workable solutions, to intractable health issues [6,7]. Transdisciplinary approaches recognise that health problems arise from highly complex interacting systems, being the combined result of multiple, intersecting variables from genetics and physiology to those manifesting from social, political and ecological causes [8]. In this paper, utilisation of this approach enabled identification and subsequent analysis of literature across a wide-range of academic disciplines and perspectives. This in turn enabled clarification of what is currently documented about the interrelationships between hunting, and the impacts on Indigenous peoples’ health inequities, including how they relate to the physical environments in which these activities are undertaken. From these analyses a new conceptual model is developed that, we argue, may enable more nuanced engagement on this complex topic, and guide enquiry and novel responses to these issues. 

It is acknowledged that “transdisciplinary” is a term used in ecohealth contexts to denote the creation of new ways of thinking through transcendence of previous discipline-specific boundaries and “ways of knowing”.

While the Ecohealth approach embraces the tools of core domains such as public health, ecology and ecosystem management, its emphasis on interdisciplinarity and cross-sectoral collaboration enables it to transcend important limits and blind spots of individual fields of expertise.

[9] (p. 2)

It is, in particular, these “blind spots” in current discipline-oriented thinking about Indigenous land-health interrelationships that this paper seeks to identify and critique.

### 1.2. Land-based Practices and Health

For many of the world’s Indigenous peoples’, the health of the land and the health of the people and community are thought to be one and the same [10,11,12].

Our identity as human beings remains tied to our land, to our cultural practices, our systems of authority and social control, our intellectual traditions, our concepts of spirituality, and to our systems of resource ownership and exchange. Destroy this relationship and you damage—sometimes irrevocably—individual human beings and their health.

[13] (p. 15)

These “ties to the land” involve an ongoing ability to engage in LBP. These include activities associated with hunting, fishing, plant cultivating and collecting/harvesting, land/water care and natural resource management, and ceremony. Numerous examples from around the world have promoted the benefits of these land ties, including:

- Human health and wellbeing, e.g., physical and nutritional benefits of catching and consuming traditional country foods, such as in Inuit communities in northern Canada [14];
- Cultural and political, e.g., the cultural pride and positive identity politics of land tenure over traditional lands, such as the sacred groves of North Pare Mountains in northeastern Tanzania [15];
- Economic, e.g., establishment of sustainable livelihood initiatives based on traditional ecosystem management practices such as harvesting wild rooibos tea by the Khoisan community in the Western Cape, South Africa [16]; and,
- Environmental, e.g., sustainable land management practices such as the Aboriginal fire abatement program in West Arnhem Land, Australia [17].

A focus on benefits is understandable but raises the question as to whether such an approach is sufficient to inform sustainable policy and program responses. There are also well-documented risks associated with aspects of Indigenous LBP participation, and the consequent adverse outcomes for human health, and the health of the environment in which these activities take place. These risks and benefits are more often presented separately and by different disciplines in the international literature. Without the availability of interdisciplinary review papers the conclusions in these separate literatures paint an inaccurate picture of the variables that need to be considered when seeking to understand the complexity of land-human health interrelationships in the Indigenous context. 

## 2. Methods

A literature review was done to identify publications focusing on Indigenous land-health interrelationships. Given the breadth of literature being considered, a wide search of electronic databases was undertaken seeking both peer-reviewed publications and published reports between 1980 and 2013 available in English. Peer-reviewed literature was identified through eight electronic library databases (CINAHL, Pubmed, Sage Journals online, Scholars Portal Journals, ProQuest Group, MEDLINE, EMBASE, and PsychINFO). The key search terms used were “Indigenous, Aboriginal, Inuit, First Nation, Native peoples, land, land-based practices, caring for country, country foods, traditional foods, bush foods, wild foods, subsistence, nutrition, food security, human health, wellbeing, culture, traditional, traditional knowledge, hunting, fishing, climate change, environmental contaminants, land tenure, tenure insecurity, health and place, vulnerability, safety”, and their variations and combinations. Published reports were identified using both online search engines and key Indigenous-focused health and environment agency websites in Australia, Canada, New Zealand and the United States. 

## 3. Results

Over 1100 publications were initially identified. Further sorting was then done to identify those publications that reported on Indigenous participation in LBP, particularly, but not limited to, hunting/fishing/collecting activities, and had explored or discussed an aspect of human health and/or wellbeing. There were 256 papers or reports subsequently identified that discussed issues and/or presented research on various aspects of these interrelationships retained for the analysis.

The vast majority of reviewed literature focused on Indigenous hunting/fishing/gathering activities in northern parts of Canada and the United States of America, with some, but to a lesser extent, looking at issues in remote northern Australia. Arctic Canada was particularly strongly represented, with an emphasis on activities associated with land and/or sea-based hunting activities. While there are findings from a review of that literature particular to the Arctic context, many of the key underlying issues and intersecting complexities identified in that literature were common to literature from other environments that shared the central themes of cumulative post-colonial consequences, competing land uses, rapid socio-economic change, diminishing intergenerational knowledge transfer, climate change impacts, and resulting “health” inequities. Interestingly, the literature coming from “the north” tended to take a more risk focused approach *versus* that from the “south” where benefits were the most commonly presented focus. Although beyond the scope of this paper, this raises intriguing issues as to why this might be the case, and acknowledges that understandings of LBP occur in cultural, not just, physical ecosystems, with multiple perspectives and influences at play. In part, this could reflect the current position of Indigenous issues within the socio-political landscape of the countries in question, whereby some emphasise the key “similarities” shared by Indigenous and non-Indigenous people’s living in that place as opposed to “differences” that Indigeneity brings to understandings of land-health interrelationships.

Appreciably, in a literature as broad as that discussing these topics in different locations, the disciplines represented in the selected publications were diverse, highlighting the inherently complex nature of the issues involved in Indigenous LBP and human-environmental health. This was a major challenge in reviewing the identified literature because the writings ranged across the fields of health and social policy, human rights and civil law, medical and health anthropology, health economics, public health, human ecology, nutritional epidemiology, Indigenous studies, environmental science, political science, climate science, nutrition and dietetics, social epidemiology, health and medical geography, sociology and psychology. 

**Figure 1:**
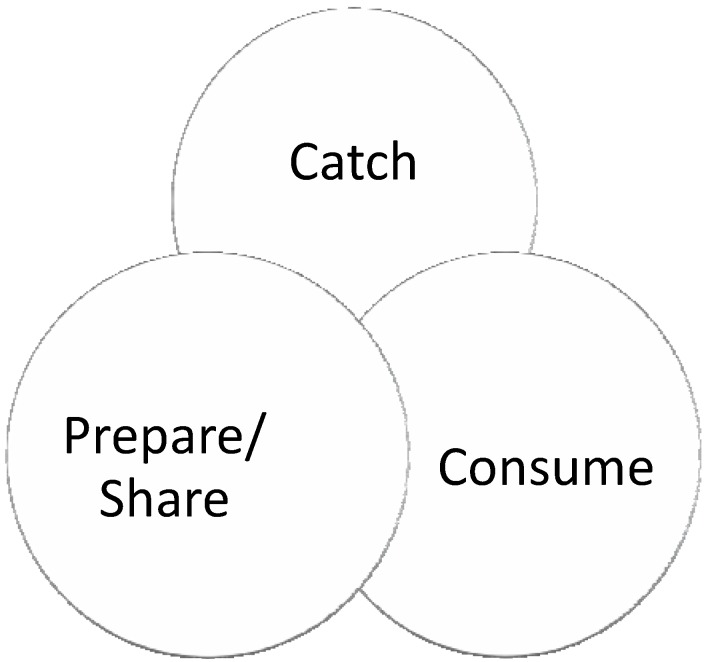
Three main sub-groupings of hunting/fishing/gathering activities associated with Indigenous participation in land-based practices with implications for human health.

Despite the diverse starting point of this review, and the varying representations of the current place and understandings of Indigenous land-health interrelationships in different ecozone contexts, two primary themes emerged from this seemingly disparate, wide spread literature. That is, the “benefits” of Indigenous people’s participation in LBP (subsistence hunting, fishing and collecting), and the “risks” participation presents for Indigenous health. A pattern also existed in the way the literature discussed these relationships. This was based on an intersecting discourse that emphasised a specific set of activities that are embedded in Indigenous peoples relationship with food accessed from the local environment. These activities relate to actions involved in three distinct but inter-related subgroups of practices—“catching”, “preparing/sharing”, and “consuming” these foods (see Figure 1). Publications reviewed predominantly focused on one of, or one specific aspect within these practices, often in isolation from associated activities within and/or between practices. The consequences of this tendency to focus on one aspect, and to do so from within a bounded disciplinary perspective, were significant. Key conflicts and important gaps were overlooked. These are argued to be the “blind spots” of concern raised by the transdisciplinary researchers, and are the main focus of this review. 

“Catch”, refers to those activities involved in the act of obtaining traditional/country/bush/wild foods (foods from the local natural environment via a number of means). “Prepare/share” involves those activities associated with preparing and/or sharing country foods that are “caught” (both giving and receiving). “Consume” refers to activities involved in eating country foods that are “caught” or received via “sharing”. Surrounding these three intersecting groups of activities are the fluctuating influences of culture, community, environment, politics, and economics. 

**Table 1:** Summary of the key benefits and risks of Indigenous participation in hunting/fishing/gathering identified in the reviewed literature.

**Benefits**	Nutritional benefits of eating country foods Physical activity associated health benefits of LBP participation Social capital gains for individuals and communities (e.g., community cohesion and connectedness, heightened wellbeing, strong cultural ties) Economic benefits for individuals and communities Environmental benefits from land care associated with LBP Food security through nutritious, readily available, supplementation of weekly diet with country foods
**Risks**	Unsafe environments (environmental hazards) caused by climate related changes e.g., thinner ice, higher UV radiation, more extreme weather events Increased risk of accidents when out on the land: ○ inherent with hunting activities, especially when using motorised transport and firearms ○ minimal use of personal safety equipment ○ associated with inadequate knowledge transfer to safely read and navigate the local environment ○ over-reliance on technology (such as GPS) to navigate ○ inadequate mechanical repair knowledge of equipment (such as snowmobiles) Exposure to environmental contaminants in consumed country foods (e.g., heavy metals and organochlorines in sea mammals) Biological contamination from emerging zoonotics, or inappropriate storage and preparation of country foods, especially associated with the loss of “traditional” food preparation practices (e.g., fermented meats among Inuit) Relying on limited diversity of seasonally variable and/or diminishing country food stocks for food security Resource depletion from over-hunting/fishing to maintain adequate food supply Prohibitive cost of equipment and maintenance required for LBP participation

LBP: Land-based practices.

### 3.1. The Benefits and Risks of LBP

Within the identified literature, the “benefits” and “risks” attributed to Indigenous LBP were synthesized and summarized, and are presented in Table 1. This initial division clearly highlights tensions and complexities regarding the net balance of health benefits contributed to Indigenous peoples by LBP. It also reflects the strong presence of the Arctic literature with its tendency to focus on hunting. On this background, and given the dominance of this ecozone-specific literature, this paper utilises hunting as an illustrative case study to explore current understandings of Indigenous land-human health interrelationships. As such, the findings are not proffered as a definitive but rather as a “window into” an intriguing, complex and highly contested space. The resulting lens provided by the “catch, prepare/share, consume” approach is subsequently used to examine how the reviewed literature has explored these issues, the potential problems with the way this exploration has been done and presented, and the tensions and complexities that result.

### 3.2. Catch

Safe and successful “catch” activities require skills, knowledge, time, equipment, physical ability, and motivation. “Catching” also requires the environment in which these activities are undertaken to be accessible, adequately stocked with animals and other collected/harvested foods stuffs, and predictable. Therefore, “catching” involves a suite of interrelated activities and circumstances that are environmental, cultural, technological, economic, educational, and human resource dependent. Despite this interrelatedness, the reviewed literature demonstrated the disciplinary bounded tendency to separate these activities/issues, and focus on only one or a few without mention or reference to the others. The result is the potential for incomplete conclusions to be drawn about the “catch” aspects of land-health interrelationships. 

#### 3.2.1. Physical Activity

According to the reviewed literature, it appeared to be a given that engaging in the “catch” aspects of LBP meant people were more physically active. This was then proffered as a major reason for promoting such activities in Indigenous populations where high levels of obesity, diabetes and cardiovascular disease often predominated [5,18,19,20]. However, few publications had actually investigated physical activity levels associated with LBP, and the papers found only presented self-report as the measure [3,21,22]. There were no quantitative studies identified. Several qualitative papers focused on the Canadian Arctic suggested that increased use of motorised transport (such as snowmobiles) had contributed to reduced levels of physical activity by Indigenous members of communities, particularly younger members [14,21,23,24]. No publication was found that specified the actual physical activities involved in “catching” country foods, nor their frequency, intensity or duration.

#### 3.2.2. Social Capital

The positive relationships between social capital and human health are well documented [25,26,27,28,29]. Social capital and “catch” activities were seen as strongly tied *i.e.*, participation in “traditional” activities, such as hunting, were considered to give people a sense of connection to their culture, environment and community. In many respects, social capital was considered to be at the heart of Indigenous ways of experiencing “health” [11,30,31,32]. In the reviewed literature, social capital was a commonly asserted benefit of LBP participation, especially the “catch” aspects because of their positive contribution to maintenance of healthy social relations [12,33,34,35,36]. 

However, few of the reviewed publications acknowledged the unequal nature of social capital experiences related to “catch” activities in Indigenous communities. Older men were seen as the most likely to engage in regular hunting/fishing activities [14,37,38], even though they were often the significant minority in communities where the majority of people were young, a common population age profile in contemporary Indigenous contexts. Those who actually did the “catching” were afforded higher status, as their hunting abilities enabled them to provide food for their families, and their skills and knowledge were considered both key to their own sense of self, and the cultural integrity of the community through the maintenance of “traditional practices” [39,40]. However, other literature suggested that if a person was a “frequent” or “heavy user” of country foods by virtue of “catching” them, they could also be the most economically, and consequently socially, marginalised because their “catching” activities were done from necessity, *i.e.*, it was a survival strategy in the absence of other ways of making a living [37]. Additionally, other than mentioning the food security disadvantage of not having a hunter in the household, particularly for women [41,42], the reviewed literature did not further explore the consequences of these potential inequities between hunters and non-hunters on broader individual and community social capital factors. 

Given the mostly positive angle many authors took on the relationship between social capital, human health, and “catch” activities, a noted contradiction was that between reported high levels of social support in Indigenous communities [43], and the often correspondingly disproportionate levels of morbidity and mortality in these settings influenced by social pathologies, such as family violence, sexual abuse, poverty and suicide [44]. Raised in the Canadian First Nation and Inuit context [43], this led to questions about the complexities between self-report of strong social relations (often connected to “being on the land”, *i.e.*, engaging in land-based practice activities) and highly “unhealthy” communities. To better understand this, some authors called for a more holistic approach to Aboriginal health in which the health of individuals is connected to the health status and behaviours of their families and communities [44,45,46,47]. This matched the literature that called for an embracing of “Indigenous constructions of health”, which emphasised the importance of the social systems within which an individual resides, and the centrality of “nature”, family and community in balance [12,48,49]. However, it could be argued that this “in balance” view assumed that “catching” activities in Indigenous communities were still possible, facilitated equity in and between community members, and were actively chosen as a means of remaining connected with “nature” and community. As this interdisciplinary review of the literature highlights, this may not necessarily be the case.

#### 3.2.3. Human Safety and Security in the Environment

Beyond rapidly changing socio-cultural and economic environments, the other major reported change is climate. Increasingly, these climate-related changes have been connected to “catch” activities. According to the reviewed literature, the most studied peoples in this regard have been Aboriginal communities in Northern Canada [50,51,52,53,54,55,56]. 

Climate change is seen as a major challenge to “catch” in the context of sustainable, and safe, Indigenous livelihoods. To “catch” country foods, people have to venture out into the environment. Given that hunting and fishing activities in these remote settings are already considered innately risky [37,54], the increased risks from climate-mediated changes are considerable [52,55]. In the Arctic regions, the “catch” associated risks linked with climate-related changes impact how and when people can safely access the land and sea to hunt/fish/gather. These impacts include decreases in ice distribution, stability and coverage; changes in snow composition; unpredictable and more extreme weather events; sea level rise; lower inland waterway levels (impacting freshwater access); permafrost thaw with associated structural instability; changes in animal travel/migration routes; increases in UV-B exposure, and numbers of mosquitos and other biting insects [54,56]. 

The research focused on climate change adaptation also identified problematic issues with respect to “catch”. On the one hand, increased use of motorised transport (such as snowmobiles, tractor-trucks, long-range boat engines), use of GPS navigation, VHF radios, and consulting satellite images of sea ice, have the potential to make hunting/fishing activities safer [54]. On the other hand, these technologies have also been reported to increase risk because they can create false confidence in the users, and people do not always have the knowledge to fix them if they fail or do not know how to “read” the landscape without them [54,56]. Compounding this are findings that use of such technology creates inequalities in communities because not everyone can afford them. This not only impacts their LBP activities and potential yield in competition with those that can, but also strains social relations [37,54]. 

Additionally, these increased risks impacted people’s ability to manage and adopt flexible approaches to resource use [57,58,59]. Arctic Indigenous hunters, for example, have traditionally been very adept at dealing with very dynamic environments [51,60,61]. However, the rapid nature of the current climate-mediated environmental changes, over-reliance on technology, and diminishing land-based hunting knowledge/skills particularly among younger hunters, have weakened Indigenous community capacities to employ these previously relied upon adaptive approaches [37,54]. 

Decades of colonisation resulted in many Indigenous communities being moved from semi-nomadic lifestyles to permanent settlements [62,63]. Although some, predominantly older, members of these communities continue to engage in LBP, the increasing move to a more market-based economy has seen an associated decreasing reliance on “traditional” activities [64,65,66]. A number of these settlements only occurred in the past five or six decades, and when people were moved from their “traditional” environments, their existing hunting skills and knowledge where not always useful in their new environment, compromising access to country foods [54,65]. Thus, “catch” skills/knowledge were location specific and not readily transferrable when environments changed.

#### 3.2.4. Connection between People and Place

The reviewed literature reported a sense amongst older Indigenous community members that the skill and knowledge required of hunting/fishing activities was rapidly diminishing [67,68,69]. Common reasons for this included the following [14,70,71,72,73]:

- Inadequate training in the techniques of hunting and fishing due to the requirements of western-style schooling, changing lifestyle aspirations;
- Lack of access to necessary funds for the purchase of capital equipment used in hunting/fishing;
- Lack of interest in becoming involved in activities that have become increasingly marginalised from an economic perspective (e.g., demise of the seal fur trade);
- Language differences now exist between generations;
- An increasing dependence upon wage employment that severely limits time available to hunt, fish and gather.

Changing social relations were also seen as a contributor, whereby a loss of respect for and/or diminishing access to elders by younger members of the community substantially reduced skills transfer [20,43]. In the reviewed literature, the importance of intergenerational knowledge transfer about how to “catch” was commonly approached as a loss of connection to land through colonisation and dispossession [74], and the consequences this had for all members of communities to engage in “traditional” land-based practices. Indeed, the adverse and ongoing impacts of colonisation featured strongly in many papers reviewed. The tension between “traditional” and “contemporary” Indigenous experiences and aspirations were commonly filtered through the pervasive lens of the legacy of colonisation. The resulting loss of connection between people and place, and the impacts on intergenerational engagement, were seen as major contributors to why “catch” activities and capacities were disappearing.

#### 3.2.5. Land Tenure

Consequences of ongoing land dispossession were strongly emphasised in the tenure insecurity literature. This literature highlighted the problems many Indigenous people faced in the context of uncertain land rights, and therefore, access to and use of, ancestral lands [75]. It found that if Indigenous people are not secure in access to and use of their lands, then resource competition can result in both conflict and indiscriminate use of resources [76]. Further, tenure insecurity has also been associated with a loss of incentive to protect the land [77], and a tendency to prioritize short-term benefits over sustainable, long-term resource use [78]. These findings are controversial given the negative light they cast on Indigenous natural resource use. However, the issue is one of tenure insecurity and its consequences for Indigenous people attempting to maintain a living in, on and from the land/sea under those circumstances. The issue clearly highlights the importance of the need to actively consider the political and legal dimensions of “catch” in these situations. 

#### 3.2.6. Natural Resource Management

The tenure insecurity literature was at odds with a substantive other literature that had investigated “catch” activities and natural resource management [1,5,79,80]. In the vast majority of cases, “catch” was seen as beneficial, for both people and place. This literature argued that Indigenous people participating in LBP provide a wide range of environmental services including border protection, quarantine, fire management, wildfire abatement, carbon sequestration and trading, weed control, feral animal control, biodiversity conservation, fisheries management, restoration of wetlands, water resource management and sustainable commercial enterprises such as eco-tourism [11,30]. Although these particular environmental benefits were being referred to in the Australian context, a number of authors articulated similar environmental gains in other geographic regions and ecosystems [81,82,83,84,85]. However, the political and legal aspects of tenure security required for sustainable access to and participation in these activities was often absent from this literature.

An extension of these reported environmental benefits were the associated human economic benefits associated with eco-tourism. Eco-tourism, which included cultural eco-tours, eco-lodges, hunting and fishing tours, cultural attractions and other nature-based facilities or services [86], was considered a particular strength of Indigenous engagement in LBP [87,88,89,90,91], particularly from a sustainable livelihoods perspective. However, on this point, there were as many cautions found in the literature about the risks of ecotourism for Indigenous people with respect to sustainable environmental management, and distribution of resources within participating communities [92,93,94,95,96,97], including the potential for a loss of authenticity for Indigenous people through engagement in these activities [98]. This last point raising the controversial issue of what “authenticity” means in the context of contemporary Indigenous lives.

This emphasis on economic benefits overrode any substantive discussion of the considerable “non-metric” gains associated with Indigenous engagement in eco-tourism related activities, such as “cultural pride” and positive identity. These issues did arise [15,16,17,88,90] but, like the point about “authenticity”, they were considerably diluted by the strong emphasis on the purported monetary and land care gains. 

#### 3.2.7. Food Security

“Catching” was also associated with food security. On the one hand, “catching” country foods was seen to potentially reduce weekly food costs *i.e.*, accessing country foods could reduce reliance on more expensive store-bought foods in remote Indigenous settings [99]. This was considered a particular issue where low household income and associated food insecurity were concerns, as was the case in a number of reported Canadian Indigenous communities [99,100,101,102]. However, in other literature, “catch” activities were associated with substantial increased costs because of the need to purchase and maintain hunting/fishing equipment (including guns and ammunition, outdoor gear and navigation equipment, motorised transport such as snowmobiles, boats or trucks, and fuel costs), as well as the costs associated with lost income from waged employment whilst away hunting or fishing [64,72,103]. 

A further reported “cost” of “catch” activities were the potential environmental consequences of depleted country foods stocks. In the Canadian Arctic, it has been widely reported that caribou stocks are diminishing. This is a popular country food source for many Indigenous people living in this region. If stock numbers are falling as suggested, the implications on food security in settings where subsistence hunting is relied upon would be considerable. With fewer animals available, despite “catch quotas” put in place for conservation reasons, over-hunting is a potential issue. Hunters would also potentially have to travel much greater distances in order to “catch” available stock, thereby adding to the costs of participation (economic and time), and increasing their risks to environmental hazards. Added to this is the finding that substituting one country food, due to reduced availability, with another that may be more available/plentiful poses challenges. There is the issue of skills/knowledge and capacity (including equipment) required to successfully hunt one species *versus* another [54]. A further issue is that of taste, which has been reported as a reason for Indigenous people often not being willing to actively substitute one species for another in their country food diet [104]. Given these factors, relying on country food sources for food security becomes questionable. 

#### 3.2.8. “Catch” in Summary

Understanding “catch” activities proved challenging from the literature reviewed, in part because the available research was strongly focused on one ecozone, the Arctic. Despite this, the many interwoven issues that comprised and informed the concept and practices of “catch” revealed a contradictory story. The social capital benefits derived from reinforcing a sense of “traditional” Indigenous identity were juxtaposed against inequities created by both gender and age, as well by who could afford “catch” equipment *versus* those that could not. Poorly supported claims of physical benefits appeared frequently, and sat in contrast to the increased physical risks posed by climate change that made an already inherently risky activity even more so. A loss of skills and knowledge in how to “catch” further added to the problem of safety, and dilution of social capital gains. Diminishing food stocks, potential for environmental exploitation, and tenure insecurity issues added further layers of complexity to “catch” activities that were not adequately considered in the more human health-focused literature. As such, “catch” becomes a challenging area in which to try to balance potential benefits and harms, many of which are not clear-cut. Based on the reviewed literature, many of these issues also do not currently appear to have a substantive evidence base to support a definitive policy position.

### 3.3. Prepare/Share

The preparation and sharing of country foods by Indigenous peoples has long been associated with maintenance of cultural traditions and strengthening of social ties [37,105,106]. These activities are considered to bond community members. However, in the reviewed literature these activities were the least evident or discussed. Few papers presented evidence or explored in any depth understandings of “prepare/share”, although statements about the significance of these activities in Indigenous communities were common. The small amount of literature that did present findings about these issues indicated several potentially conflicting perspectives with respect to both “prepare” and “share” related activities. 

#### 3.3.1. Field Dressing Techniques

One issue was that of who was actually doing the preparing. Age and gender were implied as being important with respect to this issue, although generally not articulated or critiqued. Field dressing techniques (*i.e.*, skinning, gutting, butchering, *etc.*), as well as country food preparation for both immediate consumption and storage for later consumption, require particular skills and knowledge. The nuances of these skill and knowledge sets were not clearly defined or examined in the reviewed literature. This was a major gap, which made it difficult to understand what “prepare” with respect to country foods actually involved and/or required. What was highlighted was the decline of intergenerational knowledge transfer as a major factor in the loss of this skill/knowledge set in communities [54,74]. As emphasised in the “catch” literature, this was explained by shifting social relations whereby the older community members who knew how to field dress were not able to pass their capacity on to younger community members for a variety of interrelated reasons to do with respect, language, time constraints, motivation, and costs [20,43,70,71,72,73]. Participation by younger people in waged employment and formal schooling were seen as particularly significant constraints in this regard [14,107]. These impediments were commonly cited in the reviewed literature and raise the controversial issue of how community engagement in “traditional” activities such as country food preparation sit alongside the demands of “contemporary” lives in Indigenous communities. This is particularly so for the younger generations who have grown up at a time where participation in formal schooling and waged employment have been emphasised. 

##### Food Safety

A particular point of concern raised in the literature about the consequences of these diminishing “prepare” knowledge and skills related to food safety. There was emerging evidence that “unsafe” country food preparation techniques were occurring that could lead to food contamination with pathogens such as botulism [104]. The potential significance of this was not adequately explored in the reviewed literature despite the obvious danger of handling and consuming foodstuffs contaminated in this way. As such, knowing how to “prepare” required a consideration of safe techniques. However, “safety” was only actively discussed in the “catch” and “consume” related literature. 

##### Climate and Food Preparation/Storage

Complicating this last issue were the reported impacts of warmer temperatures in settings such as the Arctic, and how these were further compromising not just food preparation but also safe country food transport and storage [55,56]. With warmer temperatures and associated unpredictable weather patterns impacting the landscape, transporting food could become unsafe, not only for those doing the transporting but also for the food itself. In addition, higher temperatures or extended periods of unseasonal warmth could compromise safe food storage [52,54,55,56]. With the speed of climate changes exceeding adaptive capacity in these settings, including the apparently yet unexplored issue of mitigation measures to address the impacts of higher temperatures on food transport and storage, the consequences could be considerable. This would have a flow on effect from the significant climate change impacts on “catch”, and further diminish safety in accessing country foods. The reviewed literature only touched on these issues with respect to “prepare” yet the implications are substantial, as highlighted by the Arctic research where these issues have been most actively explored to date.

#### 3.3.2. Sharing

With respect to “share” activities related to local food resources, the available literature indicated that the modernising of Indigenous communities had impacted sharing practices. These influences were seen to have created inequalities and impacted previous kinship-based relationships, which meant people were not sharing as much as they might have in the past. The major contributing factors were reported to be [54,107,108,109,110,111,112]:

- The introduction of waged-based economies, and the resulting differential in household incomes and time available to participate in LBP;
- Compulsory schooling, and the resulting loss of time and skills/knowledge regarding LBP;
- Contemporary lifestyles with the associated diminishing interest in “traditional” practices, especially by the young, and the introduction and increasing consumption of store-bought foods;
- Unequal purchasing capacity and, therefore, access to resources, for hunting/fishing.

These changes were noted to be complex, and varied regionally. However, there was a general sense that “share” activities were not necessarily a reliable means of ensuring food security in these settings. If this is the case, then the often repeated statement that “sharing” is a fundamental aspect of Indigenous community life, and it can be relied on to facilitate distribution of food, especially country foods, may need to be revisited as it may be undergoing transformation in some locations. Once again, the issues are complex and the available literature offered a limited view. Economic differences were highlighted, as were changing preferences. These were also mentioned in the literature relating to “catch” and aspects of “prepare” related activities. However, the breadth and depth of these intersecting factors remained largely unexplored.

##### Food Support Programs

An interesting case in point regarding this complexity was the community freezer programs that had progressively been introduced into northern Canadian Indigenous communities since the 1970s. The principle behind the community freezers in these remote, Inuit and First Nations, communities was to enable greater access to country foods. The idea was that local hunters would procure country foods that could be placed in the freezer for community use. Elders and single mothers were seen as being in particular need in these communities as they often would not have a hunter in their household or access to other means to ensure a regular supply of country foods [113]. However, it has also been noted that food availability, quality and supply are not sufficient, in themselves, to determine food security because other factors such as social relations and structure; the impact of age, gender and education level on food choices; and cultural appropriateness of available food also come in to play [114,115,116]. 

A recent community freezer-focused study undertaken in one Inuit community in northern Canada (Nain, Nunatsiavut) [113], highlighted some of these “share” related issues. Echoing other study findings [51,54,117,118] this study revealed that there was a perception that country food consumption behaviours were becoming more individualised, with a focus on only supplying to family members because of the costs of hunting (equipment, time and safety), and available yield. The community freezer therefore made it possible for community members without the means to “catch”, and those outside “share” networks, to access country foods, especially larger, more preferred, country foods such as caribou, at the time of the study. However, with the previously mentioned issue of reported caribou herd decline in these regions, the pressures of filling the community freezer to meet this need was seen as a potential contributor to over harvesting of some species. The issue of stigma attached to accessing the community freezer for those other than elders, single mothers or the sick/infirm, was not discussed. Nor was the potential for a community freezer program to disrupt previously established, and potentially highly political and adaptive, community food sharing arrangements; an issue raised in other related literature [37]. 

#### 3.3.3. “Prepare/Share” in Summary

It was noteworthy that the issues of “prepare” and “share”, although often mentioned in the reviewed literature as important to Indigenous community wellbeing, were not substantively studied. Once again, complexities became apparent when considering the role and place of “preparing” and “sharing” activities in these settings as authors tended to adopt one or other side of the more established “benefits”* versus* “risks” approach, raising questions as to the utility of these often limited perspectives. The scant literature that was identified, and the strong focus on Artic settings, made it difficult to draw any clear conclusions about how and to what extent “prepare” and “share” activities actually operate in contemporary Indigenous contexts. Being interested in and knowing how to “prepare” country foods, and do so safely, is one issue. Being willing and able to “share” what is “caught” and “prepared” is another, and these issues intersect in important ways existing literature does not appear to have considered.

### 3.4. Consume

Eating country foods has generally been considered a healthy option. Advocates across the board have emphasised the benefits for Indigenous people of “consuming” foods they procure from their local environments. Country food consumption also has strong associations with Indigenous identity, especially as challenges to “traditional” ways of life encroach into rural and remote Indigenous settings and impact bio-psycho-social health and wellbeing. Not least of these impacts has been the rise of chronic, lifestyle related health problems, such as diabetes, obesity and cardiovascular disease that are occurring in many of the worlds Indigenous populations at rates disproportionate to non-Indigenous populations in the same state/country. Additionally, social-emotional health issues, which are not separated by Indigenous peoples from their physical health, are seen to benefit from the activities associated with country food consumption. Perhaps in response to these two key issues, the most substantive literature identified regarding LBP and Indigenous health was that related to the “consume” aspects of country foods.

#### 3.4.1. “Healthy” Foods

By far the most substantive quantitative literature available on the topics of Indigenous land-health interrelationships relates to “consume” activities. There were strong arguments advocating consumption of country foods based on their high nutritional content, especially in contexts where the incidence of obesity, diabetes, cardiovascular disease and their associated adverse health outcomes are increasing. In the Arctic, substantial effort had gone into measuring the various macro and micro nutrient content of country foods [119,120,121,122,123,124,125]. Findings from these studies were often reported in the context of identified nutritional deficiencies in sub-groupings of Indigenous peoples (e.g., women of child-bearing age, children, elders), identified in food frequency questionnaires, 24 h dietary recalls [126,127], and anthropometry [128], which were attributed to a shift in diet away from “traditional” food sources to store-bought foods. By all accounts, “consuming” country foods was considered an excellent option and, therefore, should be actively promoted.

#### 3.4.2. “Contaminated” Foods

However, paralleling this was a growing literature on the presence of environmental contaminants in country foods. Concentrated on Arctic regions again, this literature highlighted the contemporary complexities of “consume” in the presence of environmental contaminants in country foods, which included mercury, cadmium, Persistent Organic Pollutants (POPs), PCBs (a group of anthropogenic industrial organochlorine chemicals) [129,130], and previously lead from lead shot fragments found in hunted game birds [131]. This was a substantive, rigorous and rapidly growing body of literature, with varying degrees of corresponding evidence presented about current understandings on how these agents impact human health when consumed in foodstuffs [130,131,132,133,134,135,136,137,138,139]. Interestingly, although the presence of these contaminants highlighted the necessity of considering land and sea use issues more broadly than just the foods themselves, the up and down stream causative chain was more implied than actively discussed in this literature. This could have been a consequence of the disciplinary bounded space in which this research was taking place and/or a reluctance to “politicise” the science. Either way, an absence of detailed discussion of the factors contributing to the introduction of these contaminants into the food chain, and a focus on the human health consequences, was apparent. Although there were indications in the literature that levels of many of these contaminants were decreasing in country foods, and thereby, also in the people that consumed them in these regions, it is important to note these contaminants can bio-accumulate in the food chain, meaning humans regularly “consuming” country foods are most affected. This is not an inconsiderable issue when the risks and benefits of increased country food consumption are under discussion.

#### 3.4.3. “Preferred” Foods

Compounding the “consume” issue further are changing food preferences. With modernising lifestyles, and with the challenges of accessing adequate amounts, and quality, of preferred country foods, many Indigenous community members are increasing their consumption of store-bought foods [109,140,141,142]. These foods are generally considered less nutritious and people, especially the young, have been reported to be trending away from country foods towards the high fat and sugar options such as carbonated drinks and snack foods [14,107]. Although a number of authors reported Indigenous peoples across the age spectrum stating they considered country foods as the most healthy, this did not necessarily translate into increased or preferential consumption of country foods [72,104,138,139,140,141,142]. 

#### 3.4.4. “Consume” in Summary

What people choose to eat is a major issue of concern for health researchers and agencies around the world [143], not just for Indigenous people. However, the life expectancy differences between Indigenous and non-Indigenous people in many of the countries to which the reviewed literature relates, made food choices and preferences a key focus because of the link with obesity and preventable cardiovascular disease [3,139,144]. This was frequently cited as the major justification for Indigenous people to consume country foods, and there was a strong body of literature that provided the nutritional evidence base to support this promotion. In contrast, important caveats to the safety of such consumption arose in the environmental contaminants literature, and the literature on changing food preferences by Indigenous people, especially the young, added further complexity to the issue of “consume” in these settings. Links between these contrasting issues were not routinely made in the reviewed literature. This posed challenges in meaningfully interpreting how the consumption of country foods actually relates to human health, and the broader implications for the health of the environment in which many of these foods are being sourced.

## 4. Discussion

The interrelationships between LBP and human-environmental health are complex. Given its strong research presence in the literature, hunting was utilised as a contemporary lens to explore understandings of these connections, and proved an intriguing case in point. This international, interdisciplinary review demonstrates the often discipline specific, fragmented and deconstructed patchwork of current understanding on this topic. The result is an uncovering of the considerable gaps, contradictions and biases that exist if one were to be interested in the central issue, that of the connections between Indigenous health and participation in LBPs. Food security; the contemporary place of cultural traditions; impacts of a market economy, waged employment and formal schooling; modernising lifestyles and changing “tastes”; land tenure; contaminants; intergenerational shifts; climate changes; identity politics including age, gender, and what it means to be “Indigenous”; cultural constructions of “health”; nutrition; and technology, all play a part. The question “is hunting still healthy?” a presumably direct question in the context of Indigenous health today, is therefore, not straightforward. 

The challenge, as always, in attempting to present evidence-based complexity from the literature is one of depth, as much as breadth. The tendency for the North American, particularly the dominant Arctic, literature to focus on “risks”, and the other literatures, especially the Australian work, to focus on “benefits”, with respect to land-human health interrelationships was intriguing. Perhaps this arose from differing “cultural” and “political” constructions and interpretations afforded these issues in these regions. It could be a consequence of how Indigeneity is currently understood and located in these societies, based on accumulated historical learning, or perhaps a tendency to emphasise “difference” over “similarities” as an extension of these politics. Alternatively, some of the identified gaps could be the result of a rejection of research by some Indigenous groups because of previous and ongoing exploitation using these techniques. Finally, it could be related to unconscious biases and assumptions arsing from deeply embedded constructions of “health” that exist in both Indigenous and non-Indigenous contexts in these locations. 

Each of these issues requires careful and lengthy exploration and discussion, and are beyond the scope of this paper. However, a strength of this paper, despite the unequal representation of research in different Indigenous contexts evident in the literature and reflected in this review, and only brief reference to some of these overarching “political” issues, is the attempt to take and critique the available literature internationally, and present it through a transdisciplinary lens. This includes identification of the significant gaps, and differences of emphasis in various ecozones, and the challenge of identifying and critiquing literature across such a diverse array of disciplines.

Despite these important caveats, the process of bringing diverse disciplinary literatures related to the same topic under the umbrella of a single paper demonstrates the challenges, importance and utility, of the insights that can emerge from gathering and reflecting on this diversity. Although hunting is only one of the many LBPs engaged in by Indigenous people around the world, it is a substantive and highly complex activity. In this way it draws together many of the major elements of LBP in Indigenous contexts and served as a useful case study to explore these complexities. 

In the context of Indigenous land-health interrelationships, the more familiar benefits *versus* risks approach to understanding the interplay of these people-place interactions only goes so far, and is not adequate to map and explore the multi-faceted realities that shape these relationships. As demonstrated through this literature review, a disciplinary-bounded lens is similarly limited. In contrast, the use of a transdisciplinary approach enabled the catch, prepare/share, consume model to emerge, with the resulting matrix-view of LBP and human-environmental health revealing a very complex picture. Although catch, prepare/share, and consume were predominantly explored using hunting, reflecting the focus in the available literature, this activity-based analysis enabled a highly nuanced understanding to emerge about what was known about these fundamentally interrelated, and socio-culturally located, aspects of this major form of LBP. Identifying and then attempting to use the catch, prepare/share, consume model quickly revealed some significant gaps in current understandings about what LBPs actually involved, and why that is important to address. 

**Figure 2:**
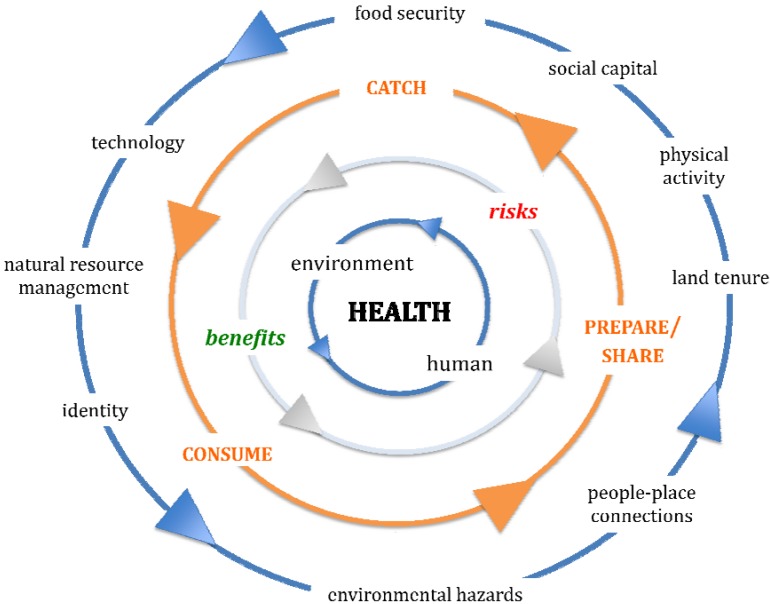
Conceptual model of the interrelationships and key drivers associated with catch, prepare/share and consume aspects of Indigenous land-based practices.

Reflecting on this initial construction, this paper sought to go further. By using the presented critique to reframe this simpler activity-focused model, a subsequent considerably more inclusive and comprehensive model is proposed. This model acknowledges the interdependence and overlap of the issues involved, and the array of factors influencing Indigenous people’s participation in LBPs, including constructions of “health”. Figure 2 presents this reframing and offers a means of engaging with the health of both people and place within this complexity. The central driver in all of the interrelationships is the environment. In this way, “health” is not separated from its social, ecological, cultural, economic, technological or political influences nor is it just focused on biological constructions of human wellbeing. This is far more in keeping with many Indigenous understandings of “health”, and challenges the reductionist tendencies of more Western representations of health where individual human biology is focused on in relative isolation from the multitude of factors that influence it. The activities of catch, prepare/share and consume are thus located in these broader influences, beyond the narrow view of this individual human biology, and out into the complex world of intersecting behaviours, politics, economies, technologies, hopes, fears, aspirations, cultures, weather, industry, and the multitude of other factors that determine benefits and risks. These all change over time, and are interpreted according to perspective, which further complicates how, what, why, when and through whom these intersections occur and are understood. 

Consideration of these overlaps, differing worldviews of “health”, power relationships, politics, and interpretations are a major consideration in this proposed model. The push/pull that occurs between these various intersections only became evident when the breadth and depth of available literature was explored, revealing the intrinsic complexity that exists.

This revised model attempts to encapsulate this inherent complexity, and offers a template for those engaged with these issues to better work in this multifaceted space. The idea is not to suggest that all of these issues be included, just that they be actively considered. Without this reflection, the “blind spots” identified in the reviewed literature will continue to hamper understandings of Indigenous land-human health interrelationships, in whatever ecozones they are studied, and result in limited or constrained solutions. 

It was utilisation of this integrated view that enabled important contradictions and omissions to be identified in this review. For example, despite the frequently reported physical activity benefits of hunting participation, these were not clarified or supported by available evidence. Equally, the vigorous promotion of country food consumption based on the nutritional evidence sat in stark contrast to the evidence about increased risks of catching or preparing it. Dangers in the environment caused by contaminants in the food chain, climate change, and profound shifts in adaptive capacities being brought about by rapid socio-cultural change, all contributed to the potential for harm. Discussion of these harms was most commonly done in literature that was separate to that emphasising the reported benefits. In the same vein, a narrow emphasis on only the human health benefits of catching, preparing/sharing and consuming country foods, was a further concern. Any gains advocated quickly become diluted, and potentially compound health inequities rather than reduce them, if the broader picture and inherent complexities surrounding these issues are not acknowledged. This tendency to emphasise the health needs of the people over the places they live was particularly worrying. “Healthy” country foods require “healthy” environments, both with respect to so called “natural habitats” as well as the social-political-economic-cultural spaces people occupy and in which they practice their activities of daily living. A failure to adequately consider this creates unease when seeking to ascertain how best to support ongoing Indigenous participation in LBPs so that people and planet can sustainably benefit.

In exploring these issues in the reviewed literature, another major problem was the general absence of substantive evidence for many of the claims made. Appreciating the opportunities and constraints of research methods to provide “holisitic” views of the issues under investigation, understanding land-human health interrelationships appeared too complex for the majority of the studies presented. The need for evidence that enables informed decisions to be made about where, and how, to focus attention is essential. However, the reliance on disciplinary-bounded investigations did not enable either the production of this required evidence nor the necessary identification and exploration of key and often contradictory intersections. With the exception of some of the consume-related literature, the vast majority of reviewed literature relied on small, qualitative studies (e.g., [14,30,52,54]). On the one hand, this approach made sense as it enabled capture of specific stories and contexts that influenced LBP and human-environment interrelationships. These are the voices of the people living in and negotiating these complex realities, and need to be recorded and heard. On the other hand, the absence of complementary quantitative data about many of the issues raised through these narratives made it difficult, if not impossible, to substantiate and subsequently argue for investment in responses to the identified key drivers impacting land-human health interrelationships. With so much at stake for both the people living in these remote and fragile environments, and the environments themselves, it could be argued that more integrated, and empirically supported research approaches are essential. 

This is not to suggest that the focus should shift to predominantly epidemiology-based research approaches. These also have their limitations, especially when seeking to elucidate the complex, culturally influenced links between health and place [145]. Questions utilising epidemiological methods are necessarily reductionist for the sake of specificity, which can then lack sensitivity and overlook context with respect to key intersecting nuances in and between areas of interest. There is a further tendency to reduce “health” to biomedical constructs such as blood pressure and serum concentrations of nutrients because these lend themselves to metrics. This cultural construction of “health”, which is Western derived, not only overlooks the bio-psycho-socio-cultural-economic-political interplays and overlaps that influence and determine health [146,147], but also takes people”s health out of the health of the places people live [148]. When considering how Indigenous LBP participation impacts “health” this is of considerable concern if such narrow tools are given preference. In this review, this was most obviously highlighted by the consume-focused literature.

The challenge is to look beyond the limitations of traditional epidemiology and bridge the reductionism of our scientific training by embracing a more ecologically focused social determinants approach to health.

[149]; (p. 469)

As such, a balance needs to be struck between the often narrow but illuminating quantitative perspectives of epidemiology, and the powerfully informative shades and nuances derived from the qualitative and mixed methods literature that add the necessary “why” and “how” understandings to the “what” of biostatistics. The linking step is then to place these collective understandings into this ecologically focused view of the social determinants of health.

If these integrated approaches offer the most potential for gaining the necessary evidence-based understandings of land-human health interrelationships, then adopting a transdisciplinary approach is the path that needs to be explored. The complex landscape that appears when disciplinary and policy fences are removed can become overwhelming. This paper seeks to present a case for active engagement with these complexities as a requirement, not an option, for development of sustainable responses. Indigenous worldviews appear to intrinsically understand this. However, perhaps key, although controversial, in approaching an issue this complex is that of the challenge of not romanticising Indigenous peoples’ land use, resource management and conservation values [150,151]. Indigenous land-health interrelationships have a long history. As with other post-settlement critiques of culture-nature relations and Indigenous politics [152,153], this paper seeks to move beyond static cultural constructions into more holistic conceptions that support Indigenous peoples ways of living now that still honour inherited responsibilities, cultural knowledge’s, and self-determination [154]. The landscapes, both “natural” and “human-made”, in which LBPs such as hunting are being undertaken requires this maturing and respectful view. Neither a single disciplinary, culturally static or methodologically limited lens provides the necessary balance or interrogation these issues demand for practical, sustainable and culturally relevant approaches to be adopted. 

In the same vein, understanding what LBP actually involves through a detailed, contemporary critique of the specifics of catch, prepare/share and consume activities and motivations, is also required. The reviewed literature demonstrated substantial holes in these understandings. Land-based practices are occurring in environments that are changing physically, socially, culturally, and economically. These changes are creating considerable tensions. Without acknowledging this and the resulting complexities, research aimed at informing policy and program responses to Indigenous health and wellbeing will inevitably fall considerably short of their intended aims. Central to this is the active consideration of the impacts of changing environments, which profoundly frame the contexts in which people are undertaking these activities. 

In this way, this review and critique of the international literature revealed some of the significant “blind spots” and limitations that are inescapable if engagement with land-human health interrelationships continues to occur in exclusively disciplinary and culturally bounded spaces.

In order to deal with these limitations, various sets of obstacles need to be revised or dismantled: first, ontological frameworks or worldviews that do not embrace the complexity of the natural and human-made environment; second, constructions of knowledge that value rational utilitarian approaches to interpret the layout, use and management of human and natural ecosystems; third, specialisation, segmentation and bureaucratization of knowledge and expertise; and finally, the lack of transfer and communication between professionals, politicians, interest groups and the public.

[155] (p. 17)

Only through such transdisciplinary-mediated approaches can the current gaps and assumptions, often incorrect, about the complex interrelationships between land and human health in Indigenous contexts become understood and sustainably addressed. 

## 5. Conclusions

“Is hunting still healthy?” is not a simple question. Despite the importance of this question in the area of Indigenous environmental health policy and programming and the context of the changing nature of Indigenous relationships with land/sea, the literature appears to have approached this question solely in a deconstructed, unidisciplinary way. Land-human health interrelationships are inherently complex. Using hunting as a powerful case in point, this paper has sought to demonstrate there is considerable benefit in looking out the window of disciplinary and culturally bounded spaces into a broader landscape in order to better understand this complexity. As unfamiliar, and potentially uncomfortable this may be its necessity is difficult to argue against. Land-based practices are neither a single act nor an entity practised in isolation of culture, society, economics, environment, politics or technology. Health of people and place demand an integrated engagement with these inherently multifarious realities. It is not viable to address one without the other, and embracing this more holistic view through development and use of new, transdisciplinary methods has considerable merit. The end goal for Indigenous people of being able to realise any benefits of ongoing LBP can only be achieved if the health of the environment is seen as integral to, not separate from, that of the people in it. Additionally, how “health” is understood requires a considerably broader lens than that focused predominantly on individual human biology. To do this requires a radical rethink in the way research and policy makers approach these complex issues.
